# Association of Asthma Control and Metered-Dose Inhaler Use Technique among Adult Asthmatic Patients Attending Outpatient Clinic, in Resource-Limited Country: A Prospective Study

**DOI:** 10.1155/2019/6934040

**Published:** 2019-08-01

**Authors:** Bezie Kebede, Girma Mamo, Abebaw Molla

**Affiliations:** ^1^Mizan-Tepi University, College of Health Sciences, Department of Pharmacy, Tepi, Ethiopia; ^2^Assistant Professor of Clinical Pharmacy, Jimma University, College of Health Sciences, School of Pharmacy, Jimma, Ethiopia; ^3^Mizan-Tepi University, College of Health Sciences, Department of Nursing, Tepi, Ethiopia

## Abstract

Asthma is a heterogeneous disease which is characterized by chronic airway inflammation. It is a common chronic respiratory disease affecting 1–18% of population in different countries. It can be treated mainly with inhaled medications in several forms, including pressurized metered-dose inhaler (MDI). Patients encountered difficulty in using inhaler devices even after repeated demonstration and/re-evaluation. This could highly compromise patient treatment outcome/asthma control. To evaluate relationship between MDI use technique and asthma control among adult asthmatic patients who attend respiratory clinic in Jimma University Medical Center (JUMC), Southwest Ethiopia. A prospective observational study was conducted from March to August 22, 2018. All adult asthmatic patients who met the inclusion criteria were included in the study. Patient baseline assessment was conducted (patient demography, inhalation technique, adherence, and asthma control status). Inhalation technique was obtained using a standard checklist of steps recommended in National Institute of Health (NIH) guidelines. Patient adherence using asthma inhalation test and asthma control status was assessed by 2017 GINA guideline. Independent predictors of outcome were identified, strength of association between dependent and independent variables was determined by using ordinal logistic regression analysis, and statistical significance was considered at *P* < 0.05. One hundred forty patients were included in the analysis. Among these, 26 (18.4%) patients were controlled, 65 (46.1%) partially controlled, and 35% uncontrolled. Proportion of patients with uncontrolled asthma were higher among inefficient as compared to efficient, whereas patients with controlled asthma were higher among efficient as compared to inefficient. Asthma control status is significantly associated with inhalation technique (*P*=0.006). Since most of the patients were inefficient and it is significantly associated with asthma control status, the hospital tried to adopt video MDI teaching program, and the patient should ask healthcare professionals how to take medication and they should bring their device to receive demonstration during visit. Health professionals should re-evaluate the patient during their hospital visit and encourage bringing their device to give demonstration.

## 1. Introduction

Asthma is a heterogeneous disease which is characterized by chronic airway inflammation. It is defined by history of patient respiratory symptoms such as wheeze, cough, shortness of breath, and chest tightness. Intensity and frequency of symptoms can vary from time to time and together with variable air flow limitation [1].

An increasing number of risk factors have been linked to the development of asthma, particularly found in developing countries. These include indoor-like biomass, outdoor pollution, and occupational exposures [2].

Based on previous reports, asthma has negative consequences, such as impairment of quality of life and reduction of daily living activities and direct and indirect costs related to emergency room visits, hospital admission, frequent outpatient visits, and school and work absenteeism [3], for both social and economic aspects.

The goal of asthma treatment is to control the frequent symptoms of asthma and improve forced expiratory volume or pulmonary function. Different medications can be used for this problem such as inhaled corticosteroids, long-acting *β*2 agonists, or both in combination are effective so as to achieve this outcome. However, achieving asthma control is not easy, and only a small number of asthma patients use the medications regularly as recommended or incorrect inhalation technique contributes for suboptimum treatment outcome [4].

A previous study showed that different factors were considered as predictors for asthma control status, such as the socioeconomic status, knowledge about the disease, and its triggering factors, poor perception about the pathophysiology of the disease/obstruction of bronchial tree, presence of comorbidities, fear of drug adverse effects, and ability to use different inhaler devices. Inappropriate inhaler devices and inefficient inhalation techniques can negatively affect the deposition of drugs in the target site (pulmonary) and increase the frequency of local and systemic adverse effects and prone to the patient with uncontrolled asthma [5].

In Brazil, among patients using an MDI without a spacer, a large number of patients made mistakes in the steps of keeping the mouthpiece at a correct distance from the lips, exhaling fully before using the device [6].

So this research will show the gab of patients' inhalation technique and its contribution for asthma control status and it can figure out the predictors. There is no study done before with this specific title in Ethiopia and so it could serve as baseline for further study.

## 2. Methods and Participants

This study was done in JUMC, Jimma, Ethiopia. JUMC is located in Jimma town which is 346 km away from Addis Ababa, and it is found in southwest Ethiopia. It is one of the largest teaching university hospitals in Ethiopia. JUMC is offering diagnosis and treatment for approximately 10,791 patients per month. There are about 9 outpatient clinics located within the hospital which serve over 9592 visits/month [7]. Among this outpatient department (OPD) visits, about 45 patients are asthmatic per month [8]. This study was conducted specifically at an OPD service, which is a respiratory clinic, from March to August 22, 2018 G.C.

A hospital-based prospective study was used to evaluate the association of asthma control and metered-dose inhaler use technique among asthmatic patients attending respiratory clinics in JUMC. All adults of age 18 and above, patients with confirmed diagnosis of asthma and on controller medications for at least the last three months, and who are willing to participate and having follow-up at the outpatient respiratory clinic were included in the study. Patients who were on exacerbation during data collection period, patients who were handicapped, and whose age was >75 years old were excluded from the study. Finally, all adult patients who satisfied the inclusion criteria were chosen as subjects for the study.

### 2.1. Selection of Study Participants

**Figure 1:**
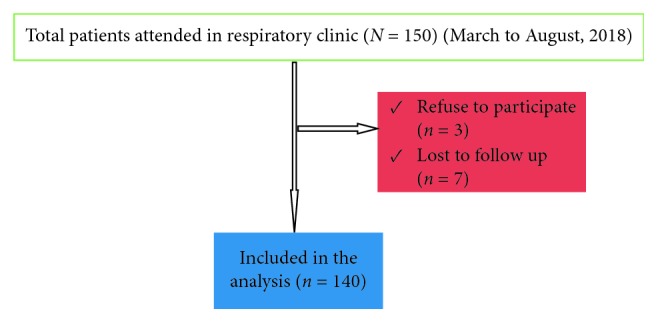
Summary of sampling procedure.

As shown in Figure 1, all adult patients who met the inclusion criteria and presented to the hospital in the data collection period were recruited in the following way.

### 2.2. Data Collection Procedure and Analysis

Relevant information like patient characteristics, inhalation technique, current medications, comorbidities, duration of illness, inhaler use, and adherence (assessed by asthma inhaler test) was recorded using a structured questionnaire (adapted from different published literatures [9–13]). A questionnaire was translated to the local language and retranslated to English. Relevant data were obtained by interviewing the patient and chart review when necessary.

An empty and their own MDI were adapted to enable a patient's inhalation technique to be recorded and patients were asked to use their aerosol just as if they would be at home. Inhalation techniques were identified using a standard checklist of steps recommended by National Institute of Health (NIH) guidelines [14] with 1 point given for each step performed correctly (maximum score = 8). Inhalation technique was dichotomized as efficient and inefficient. Patients who performed three critical steps correctly regardless of the other steps were considered as efficient and otherwise inefficient [15].

Data were collected by two pharmacists after one day training. Supplementary information and clarifications on some patient's medical information were obtained through discussion with respective nurses and physicians.

Data were entered into a computer using EpiData 3.1 software and analyzed with SPSS version 21. Before analysis, presence of collinearity between independent factors (having less than 3 variance inflation factor) and model fitness (with Hosmer–Lemeshow *P* value 0.156) was checked. Chi-square statistics was used to check adequacy of cells for ordinal logistic regression. Independent predictors of outcome and strength of association between dependent and independent variables were identified by using ordinal logistic regression analysis and *P* value <0.25 entered to multiple regression. *P*-value <0.05 was considered as significant. Descriptive statistics was used to characterize asthma control and independent variables. Results of the study were organized in the form of frequencies and percentages. The data are summarized and described using tables and figures.

## 3. Results

### 3.1. Background Characteristics of Participants

A total of 140 patients were included in the study. Of which 78 (55.7%) were females. The overall response rate was 98%. The mean age was 47.8 (age range 19–74) years with the maximum number of patients being in the age group of 41–59 years. A majority of 110 (78.57%) patients were found to have isolated asthma. Fifty-seven (40.7%) patients had moderate persistent asthma, 35% had severe persistent, and the rest (24.3%) had mild persistent. Only 3 (2.1%) of the study subjects were currently smokers while 121 (86.43%) were nonsmokers.

Seventy-seven (55%) and 72 (51.4%) patients drank alcohol and chewed khat, respectively. A majority of 88 (62.9%) patients were exposed to biomass fuels during cooking food, and other activities are shown in (Table 1). Among these, females were 55 (39.29%). The median duration of illness and MDI experience was 4 years (ranges from 4 months to 42 years) and 3 years, respectively. Sixty-nine (49.3%) had experienced an asthma exacerbation in the past 12 months. About 29.3% patients were admitted to the hospital, and only 9.76% admitted more than 2-3 times per year.

### 3.2. Inhalation Technique and Asthma Control Status

One hundred thirty (92.85%) patients had errors in one or more steps of inhaler technique. The highest number of errors was found to be done in steps inhale slowly, simultaneously press canister, and breathing in slowly and deeply which is 97 (69.28%). The second most frequent error was lean head slightly back, 69 (49.3), and take inhaler out of mouth and hold breath for 5–10 sec was the third most missed step which accounts 54 (38.6%). In this visit, the most correctly followed step was shake the inhaler vigorously (5–10 times), only 3 (2.14%) patients missed this step. Only about 26% patients were efficient and the rest were not efficient.

**Figure 2:**
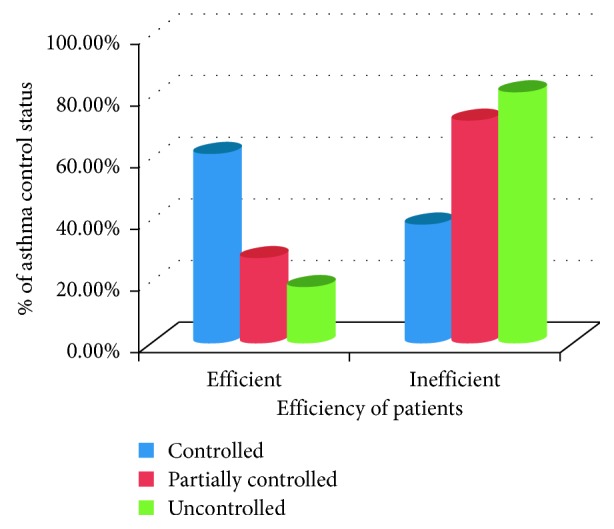
The relationship between inhalation technique and asthma control status, respiratory clinic, JUMC, Ethiopia, 2018.

As shown in Figure 2, after intervention among the total patients who had controlled asthma, 16 (61.5%) of them were efficient. Among patients who had partially controlled asthma, 72.3% patients were inefficient. Proportion of patients with uncontrolled asthma were higher among inefficient as compared to efficient, whereas patients with controlled asthma are higher among efficient as compared to inefficient. Patients with uncontrolled asthma as compared to partially controlled/controlled asthma significantly associated with inhalation technique and are more likely to use asthma devices improperly (*P*=0.006, Table 2).

**Figure 3:**
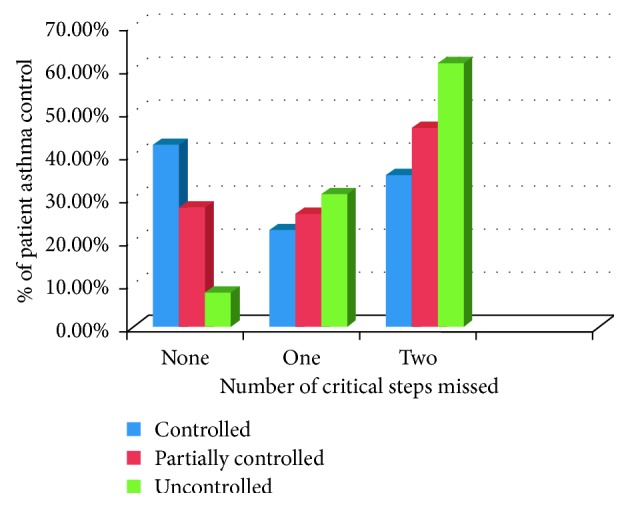
The relationship between asthma control status and number of critical steps missed, respiratory clinic, JUMC, Ethiopia, 2018.

As shown in Figure 3, among the total of efficient patients, about 42.3% of them had controlled asthma, whereas 8% of patients had uncontrolled asthma. Among the total of uncontrolled patients, 61.2% of them missed two critical steps and 30.8% missed only one critical step. The proportion of partially controlled patients was high in those who missed two critical steps (46.2%). Uncontrolled asthma as compared to partially controlled/controlled asthma was higher in those patients who missed two critical steps. In general, as the number of missed critical steps increases percentage of patients with asthma instability increased.

**Figure 4:**
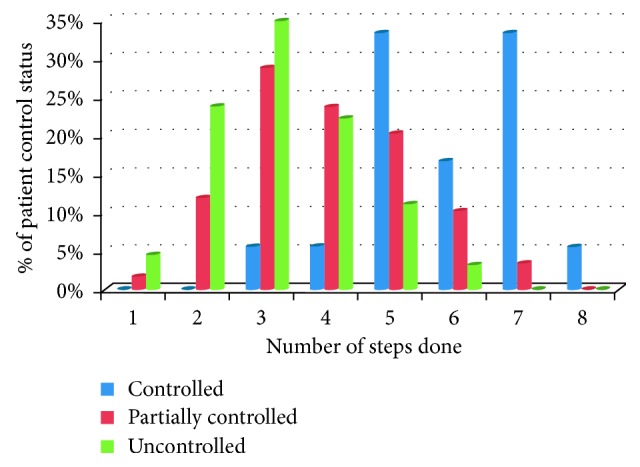
The relationship between number of steps done and asthma control status, respiratory clinic, JUMC, Ethiopia, 2018.

As shown in Figure 4, none of the patients who performed only one and two steps had controlled asthma and none of the patients had uncontrolled asthma who did more than six steps. Among all controlled patients, 88.8% of them carried out more than four steps and only 14.3% followed more than four steps among uncontrolled patients. About 33.9% partially controlled patients did more than four steps. The proportion of asthma stability was higher in those patients who carried out more than four steps.

## 4. Discussion

The proportion of patients with uncontrolled asthma was relatively higher among inefficient patients as compared to efficient, demonstrating that inappropriate use of the devices is one of the significant predictors of asthma symptom control. Correct performance of critical steps involved in using the devices was one of the factors affecting asthma symptom control. These steps which include inhaling slowly and deeply are crucial for fully delivering the drug to the lung, and, in the present study, this key step was the one with the highest proportion of errors. This might have contributed to the lack of asthma control among those who did not take a slow and deep inhalation correctly.

The proportion of patients who had daytime symptoms more than twice per week, nighttime awakening, and activity limitations were higher in our study as compared to the study revealed in Addis Ababa which showed that 62 (34.6%) patients demonstrated improper inhaler technique [16]. In our study, the proportion of inefficient patient was higher as compared to this finding. So, this higher proportion of inappropriate use of asthma device may contribute to higher proportion of asthma instability in this study and it could also be environmental difference.

Multivariate ordinal regression showed that three variables (exacerbation, adherence and inhalation technique) were identified as independent significant predictors for treatment outcome/asthma control. Efficient patients were less likely to have uncontrolled asthma as compared to inefficient (*P*=0.006). This study is consistent with the previous study done in India, Addis Ababa, Serbia, Italy, and France [15–19] which showed that inappropriate inhaler technique was associated with poor disease control and increased consumption of health care resources.

Limitation: our study relies on physician diagnosis of asthma and on small sample size.

## 5. Conclusion

This study revealed that though majority of patients claimed to know how to use inhalation devices correctly, only few patients followed all the essential steps of inhalation technique of MDI before intervention. This study showed that there is significant association between inhalation technique and asthma control. Health professionals should educate patients about inhalation technique during their hospital visit to optimize inhalation technique and intern asthma control. Exacerbation, adherence, and inhalation techniques were identified as independent significant predictors for treatment outcome/asthma control. As there are still a lot of illiterate people in our patients, the need for proper counseling during their visit is even more important. Therefore, proper and regular training about error-free inhalation techniques needs to be conducted to optimize the patient treatment outcome.

## Figures and Tables

**Table 1:** Sociodemographic and clinical characteristics of participants, respiratory clinic, JUMC, Ethiopia, 2018.

Sociodemographics and characteristics of patient	Category	Number	Percent	Mean + SD	Range
Sex	Male	62	44.3		
	Female	78	55.7		

Age group	19–40	46	32.85	47.8 ± 15	19–74
	41–59	52	37.15		
	60–75	42	30.0		

Comorbidity	Yes	30	21.43		
	No	110	78.57		

Severity of disease	Mild persistent	34	24.3		
	Moderate persistent	57	40.7		
	Sever persistent	49	35		

Residence	Urban	59	42.1		
	Rural	81	57.9		

Exposure of biomass	Yes	88	62.9		
	No	52	37.1		

Knowledge	Good	30	21.4		
	Moderate	47	33.6		
	Poor	63	45.0		

Attitude	Positive	98	70.0		
	Negative	42	30.0		

Adherence	Adherent	87	62.14		
	Intermediate	30	21.42		
	Nonadherent	23	16.44		

Previous education	Yes	60	42.85		
	No	80	57.15		

**Table 2:** Bivariate and multivariate ordinal regression analysis for predictors of poorly controlled asthma respiratory clinic, JUMC, Ethiopia, 2018.

Variables		Asthma control status (%)			Bivariate analysis		Multivariate analysis	
		Controlled	Partially controlled	Uncontrolled	CB (CI)	*P* value	AB (CI)	*P* value
Age	19–39	46.2	26.2	26.5	−0.56 (−1.36–0.26)	0.138	−0.74 (−1.58–0.113)	0.089
	40–59	30.8	43.0	40.8	−0.07 (−0.82–0.69)	0.180	−0.27 (−1.07–0.52)	0.502
	60–75	23.0	30.8	32.7	1.00	1.00	1.00	1.00

Residence	Rural	57.7	61.5	53.1	−0.196 (−0.83–0.44)	0.054	0.099 (−0.611–0.81)	0.784
	Urban	42.3	38.5	46.9	1.00	1.00	1.00	1.00

Exposure of biomass	Yes	53.8	66.2	63.3	0.19 (−0.46–0.83)	0.174	0.06 (−0.66–0.77)	0.879
	No	46.2	33.8	36.7	1.00	1.00	1.00	1.00

Adherence	Adherent	62.06	63.93	62.5	−0.64 (−2.02–0.74)	0.236	−1.21 (−2.36, −0.064)	**0.038**
	Intermediate	24.13	21.31	20.83	−0.58 (−2.07–0.92)	0.247	−1.09 (−2.122, −0.061)	**0.039**
	Nonadherent	13.81	14.76	16.67	1.00	1.00	1.00	1.00

Attitude	Positive	80.8	72.3	61.2	−0.65 (−1.34–0.044)	0.067	−0.581 (−1.31–0.15)	0.118
	Negative	19.2	27.7	38.8	1.00	1.00	1.00	1.00

Exacerbation	Yes	42.3	41.5	63.3	0.69 (0.06–1.33)	0.032	0.77 (0.11–1.43)	**0.022**
	No	57.7	58.5	36.7	1.00	1.00	1.00	1.00

Inhalation technique	Efficient	42.3	27.7	16.3	−0.89 (−1.614−(−0.17))	0.016	−1.064 (−1.83, −0.03)	**0.006**
	Inefficient	57.7	72.3	83.7	1.00	1.00	1.00	1.00

Knowledge	Good	26.92	21.54	24.5	−0.12 (−0.904–0.67)	0.775		
	Moderate	34.62	30.8	28.57	−0.218 (−0.94–0.51)	0.557		
	Poor	38.46	52.34	46.93	1.00	1.00		

Comorbidity	Yes	26.72	33.84	22.45	−0.26 (−0.95–0.43)	0.46		
	No	73.28	66.16	77.55	1.00	1.00		

Khat chewing	Yes	53.84	49.23	53.0	0.024 (−0.59–0.65)	0.94		
	No	46.16	50.77	47.0	1.00	1.00		

Alcohol drinking	Yes	46.16	60.0	53.0	0.07 (−0.55–0.69)	0.817		
	No	53.84	40.0	47.0	1.00	1.00		

Drugs other than antiasthmatic	Yes	26.9	21.5	18.4	−0.324 (−1.1–0.435)	0.403		
	No	73.1	78.5	81.6	1.00	1.00		

## Data Availability

The data used to support the findings of this study are available from the corresponding author upon request.

## Abbreviations

GINA: Global Initiative National Asthma
JUMC: Jimma University Medical Center
MDI: Metered-dose inhaler
NIH: National Institution of Health
OPD: Outpatient department
TAI: Test of asthma inhalation.
